# Life after Burn, Part II: Substance Abuse, Relationship and Living Situation of Burn Survivors

**DOI:** 10.3390/medicina58050563

**Published:** 2022-04-19

**Authors:** Christian Smolle, Maria-Fernanda Hutter, Lars-Peter Kamolz

**Affiliations:** Division of Plastic, Aesthetic and Reconstructive Surgery, Department of Sugery, Medical University of Graz, 8036 Graz, Austria; mf.hutter@gmail.com (M.-F.H.); lars.kamolz@medunigraz.at (L.-P.K.)

**Keywords:** burn injury, social reintegration, postburn, relationship, living situation, substance abuse

## Abstract

*Background and Objectives*: After burns, social reintegration is a primary long-term objective. At the same time, substance-abuse disorders are more common in burn patients. The aim of this study was to assess prevalence of substance abuse pre- and postburn as well as living situation and relationship status relative to patient-reported health-related quality of life (HRQoL). *Patients and Methods*: Burn survivors treated as inpatients between 1 January 2012 and 31 December 2019 were retrospectively identified. Collected clinical data included: age, gender, time since injury, burn extent (%TBSA), and substance abuse. Patient-reported living situation, relationship status, smoking habits, alcohol and drug consumption pre- and postburn as well as the SF-36 study were ascertained via telephone survey. Inductive statistical analysis comprised uni- and multivariate testing. A *p* < 0.05 was considered as statistically significant. *Results*: A total of 128 patients, 93 (72.7%) men, with a mean age of 40.0 ± 15.7 years were included. Mean TBSA was 9.2 ± 11.0% and significantly lower in women (*p* = 0.005). General health SF-36 scores were significantly lower in women (67.6 ± 29.8) than men (86.0 ± 20.8, *p* = 0.002). Smoking decreased from 38.8% pre- to 31.1% postburn. A significant reduction in alcohol consumption was noted over time (*p* = 0.019). The rate of never-drinkers was 18.0% pre- and 27.3% postburn. Drug abuse was rare both pre- (7.8%) and postburn (5.3%). Living situation remained stable. None of the participants depended on assisted living or lived in a care facility postburn. In total, 75.8% and 67.2% were in a relationship pre- and postburn. Patients with higher alcohol consumption postburn were significantly more often male (*p* = 0.013) and had higher SF-36 general health scores (*p* < 0.001). *Conclusions*: HRQoL is better in men than in women after burn injury. A slight decrease in substance abuse postburn was noted. The connection between HRQoL and substance abuse after burn injuries needs to be investigated further in the future.

## 1. Introduction

Even nowadays, burns remain to be common and devastating injuries with significant and sometimes long-lasting effects on burn victims’ physical and emotional well-being [1,2]. Since the evolution of modern burn care during the mid-20th century, burn treatment and goals of therapy have undergone several paradigm changes, depending on the latest clinical knowledge, available resources, and the continuous objective to meet patients’ needs in the best possible way [3]. Thus, the primary treatment goal “survival” was, by and by, augmented by the objective “quality of life”.

Since the early 2000s, burns research has increasingly focused on possibilities and measures to enhance postburn quality of life by reducing the need for immediate surgery, reduction in painful dressing changes, and optimizing fluid management in the acute setting. Furthermore, important long-term outcome parameters such as scar quality, pain and itching, post-traumatic stress disorder, and burn rehabilitation in general were increasingly addressed [4,5,6]. The outcome of these measures was assessed in several studies, of which the vast majority focused on health-related quality of life (HRQoL) and mental and physical disability assessment after burn injuries [7].

Until recently, a paucity of studies evaluated the actual impact of these measures on reintegration of burn patients into their pre-injury life. Evidence suggests that besides injury severity and physical comorbidities, substance-abuse disorders and psychiatric comorbidities have an impact on social reintegration postburn. In addition, substance abuse and psychiatric diagnoses have been observed more frequently in burn patients [8,9,10,11]. At present, it is not clear whether substance-abuse disorders are aggravated by burn injuries or not. Furthermore, the association between substance abuse and postburn HRQoL and social reintegration has not been investigated.

The aim of this study was to assess prevalence of substance abuse pre- and postburn as well as living situation and relationship status relative to patient-reported HRQoL.

## 2. Patients and Methods

### 2.1. Study Design

The study design was a single-center follow-up study on burn patients treated as inpatient cases at the Division of Plastic, Aesthetic and Reconstructive Surgery, Department of Surgery of the Medical University of Graz, Austria, between 1 January 2012 and 31 December 2019; thus covering an observation period of eight years. The telephone survey was conducted between 1 November 2020 and 5 May 2021.

### 2.2. Questionnaires

As a reference, the German version of the well-validated SF-36 HRQoL Version 1.0 questionnaire was used (English version available from www.rand.org/health-care/surveys_tools (accessed on 1 March 2022) [12,13]). This was augmented by several specific questions for relationship status, drug, alcohol and tobacco abuse, and living situation before and after the injury, respectively. A translated version of these questions is available in Supplementary Table S1.

### 2.3. Inclusion and Exclusion Criteria and Participant Recruitment

Patients that were ≥18 years at the time of injury, with burns covering any % total body surface area (TBSA) and regardless of mechanism were included in the study.

Furthermore, patients had to be able to fill out the questionnaire (i.e., no severe cognitive impairment) and had to have an Austrian phone number. Potential participants were contacted via telephone and informed about the study. If they were willing to participate, a consent form was sent via email to be returned signed by mail or email. After consent was given, the questionnaire was read to them and questions were repeated if necessary. Patients that were unwilling or unable to participate were excluded from the study.

### 2.4. Collected Clinical Data

Of those patients willing to participate, the following data was retrospectively collected from the hospital information system in a retrospective manner:

- Age at time of injury and at time of inquiry.
- Gender.
- %TBSA.
- Presence of 3rd degree burn injury.
- Inhalation injury.
- ABSI Score.
- Burned body part (head + neck, anterior trunk, back, upper extremity, hand, lower extremity, genital region).
- Days in the intensive care unit (ICU).
- Length of hospital stay (LOS).
- Psychiatric comorbidities.
- Substance abuse (tobacco, alcohol, illicit drugs).

Cases with missing data were excluded from further analysis.

### 2.5. Statistical Analysis

Statistical analysis was carried out with the program SPSS 27.0 (IBM Inc., Armonk, NY, USA) for Windows. For comparison of dichotomous variables, the Chi^2^-test was used; ordinal variables were compared using Spearman’s correlation analysis. Continuous parameters were compared using Student’s *t*-test, whereas the Levene test was used to assess for similarity of variances. Linear regression analysis was used to assess relative influence of patient-specific parameters on ordinal results of the survey. In all cases, a *p*-value < 0.05 was considered the statistical level of significance. In the following, continuous parameters are given as means plus standard deviation, whereas discrete parameters are presented as medians plus interquartile range.

## 3. Results

### 3.1. Included Participants

Of 416 inpatient cases treated for their burn injuries in the respective timeframe, ultimately 128 patients gave their consent to participate in the study, corresponding to a survey recall of 33.0%.

There were 35 (27.3%) female and 93 (72.7%) male participants. Mean age at the time of injury was 40.0 ± 15.7 years, and the mean age at the time of inquiry was 45.1 ± 16.2 years. Overall mean TBSA was 9.2 ± 11.0% and was significantly higher in men. Sixty-six patients suffered 3rd degree injuries (51.6%). The most commonly affected body area was the upper extremity (64.1%), followed by the lower extremity (53.1%) and head and neck (49.2%). Hand burns occurred in 53 (41.4%) cases, whereas the genitals were affected in a minority of patients (*n* = 6, 4.7%). Hands and head/neck burns were less common in female patients. There were 5 (3.9%) documented inhalation injuries of which all occurred in male patients. The median ABSI score was 5 (3.5–6) and was significantly lower in men (*p* = 0.021). Each patient required one surgery on average. Only 24 patients (18.8%) required intensive care and mean overall LOS was 15.5 ± 16.4 days. Significantly more men required intensive care treatment (*p* = 0.020, Table 1). The most common injury mechanism was flame burn (*n* = 71, 55.5%) followed by scald (*n* = 40, 31.3%). Most injuries happened in a domestic environment (53.1%) or were work-related (39.5%). The rest were a result of traffic accidents (*n* = 5, 3.9%), self-inflicted (*n* = 1, 0.8%), assault (*n* = 1, 0.8%), or not further specified circumstances (*n* = 2, 1.6%). There was a significant difference in the distribution of injury circumstances between men and women (Table 1).

### 3.2. Substance Abuse and Psychiatric Preconditions

In the present study collective, substance abuse and psychiatric preconditions were comparably rare. Psychiatric illnesses were documented in only 8 (6.3%) cases, whereas 13 (10.2%) patients were smokers and alcohol or drug abuse was reported in 4 (3.1%) and 2 (1.6%) cases, respectively. All but smoking was significantly more common in women (Table 2).

### 3.3. Health Related Quality of Life—Results of the SF-36 Questionnaire

**Figure 1:**
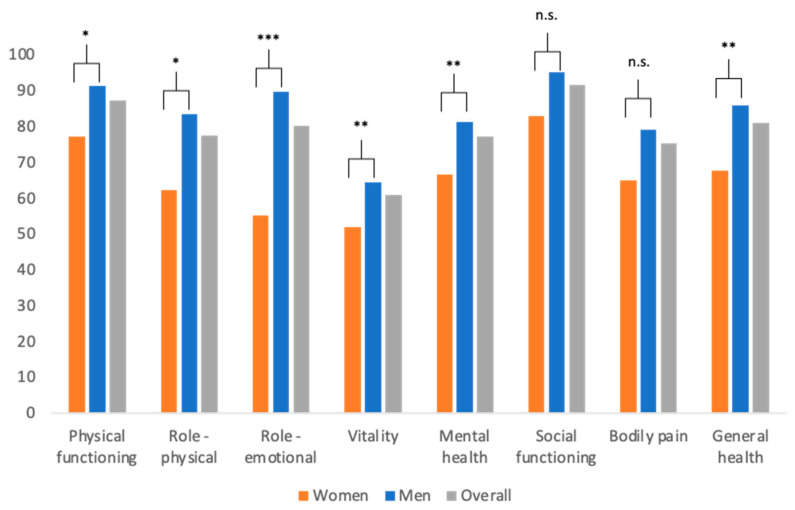
SF-36 domains and general health divided by gender and overall. Mean values ± standard deviation for each domain, given for women/men/overall (*p*-value): physical functioning 77.3 ± 29.0/91.2 ± 16.3/87.3 ± 21.4 (*p* = 0.010). Role—physical 62.1 ± 47.5/83.3 ± 35.1/77.5 ± 40.0 (*p* = 0.020). Role—emotional 55.2 ± 49.8/89.6 ± 30.3/80.2 ± 40.0 (*p* < 0.001). Vitality 51.9 ± 21.0/64.3 ± 18.1/80.9 ± 19.7 (*p* = 0.003). Mental health 66.6 ± 21.9/81.3 ± 13.7/77.3 ± 17.5 (*p* = 0.001). Social functioning 82.9 ± 33.9/95.0 ± 17.9/91.7 ± 23.9 (*p* = 0.050). Bodily pain 64.8 ± 40.3/79.2 ± 30.3/75.2 ± 33.8 (*p* = 0.061). General health 67.6 ± 29.8/86.0 ± 20.8/81.0 ± 24.9 (*p* = 0.002). n.s. = not significant, * *p* < 0.05, ** *p* < 0.01, *** *p* < 0.001.

The survey was conducted approximately 5 years after the injury on average (58.9 months in women, 62.0 months in men; overall: 61.1 months, *p* = 0.538, see Table 1). Reported general health at the time of inquiry was 81.0 ± 24.9 points and significantly higher in men (86.0 ± 20.8) than in women (67.6 ± 29.8, *p* = 0.002). Accordingly, the other seven domains of the SF-36 were divided by gender. This revealed that men scored significantly higher than women in every single one but social functioning (*p* = 0.050) and bodily pain (*p* = 0.061). The exact values and all *p*-levels are provided in Figure 1.

### 3.4. Patient-Reported Relationship Status, Living Situation, Smoking, Alcohol Consumption, and Substance Abuse Pre- and Postburn

Table 3 provides an overview on relationship status, living situation, and substance abuse pre- and postburn as reported by the survey participants. Approximately 2/5 of the study collective (42.2% pre and 41.4% postburn, respectively) were married at the time of burn injury and at the time of the survey. The number of participants in a stable relationship was lower postburn than at the time of injury (*n* = 43, 33.6% vs. *n* = 33, 25.8%)—concomitantly, the number of singles had increased from 28 (21.9%) preburn to 38 (29.7%) postburn. The number of widowed patients remained fairly stable (3 and 4, respectively). Overall, there was no significant change in relationship status from pre- to postburn (*p* = 0.415). Concerning the living situation, a slight, non-significant shift from 76 (59.4%) to 81 (63.3%) participants owning a house or apartment instead of renting was noted (*p* = 0.521). None of the survey participants reported to live in assisted living or a care facility. The number of regular smokers had decreased from 68 (53.1%) pre- to 81 (63.3%) postburn, and a decrease in smoking frequency was reported as well. None of these changes were significant though (*p* = 0.130). A significant and consistent decrease was noted concerning alcohol consumption. While the number of never-drinkers increased from 23 (18.0%) to 35 (27.3%), the number of regular drinkers (>4 times per week and 2–3 times a week) had decreased by 4.7% and 6.3%, respectively (*p* = 0.019). The number of participants reporting drug abuse was low pre- (*n* = 10, 7.8%) and postburn (*n* = 7, 5.5%, *p* = 0.451).

#### 3.4.1. Association between HRQoL and Social Reintegration Postburn

For further analysis, postburn relationship status was divided into “married/relationship” and “single/widowed” and postburn tobacco consumption was divided into “smoker” and “non-smoker”. Similarly, alcohol consumption was split into “high consumers” (2–3 ×/week and >4 ×/week) and “low consumers” (never, 1 ×/month, 2–4 ×/month). Mean SF-36 general health scores did not differ significantly between patients that were married/in relationship or singles/widowers, lived in rented or owned houses/apartments, and did not differ between smokers and non-smokers or drug-users and non-users postburn. Surprisingly though, mean SF-36 scores were significantly higher in patients reporting “high consumption” of alcohol (*p* < 0.001, see Table 4).

#### 3.4.2. Factors Associated with Decreased Reported Alcohol Consumption Postburn

For further investigation, a backwards linear regression analysis comparing the parameters age at injury, gender, %TBSA, 3rd degree burn, inhalation injury, LOS, number of surgeries, hand burns, head/neck burns, genital burns, psychiatric comorbidities, and results of the SF-36 general health section against postburn alcohol consumption was performed, as listed in Table 3. Patient-reported higher alcohol consumption postburn correlated solely with better SF-36 scores (regression coefficient 0.015, *p* < 0.001) and male gender (regression coefficient 0.570, *p* = 0.013; constant: regression coefficient −0.088, *p* = 0.790).

Finally, SF-36 scores were compared between patients with and without documented alcohol abuse: mean SF-36 general health scores were significantly lower in patients with documented alcohol abuse (40 ± 17.8 vs. 82.3 ± 24.0, *p* < 0.001).

## 4. Discussion

This study aimed to review the living situation, relationship status, and self-reported substance abuse (tobacco, alcohol, and illicit drugs) of burn survivors. Furthermore, possible associations between age, gender, injury characteristics, the results of the SF-36 questionnaire, and pre-documented psychiatric conditions or substance abuse were investigated.

With a mean age of 40 years, a gender distribution of 72% males and 28% females and a mean TBSA of 9%, our patients were comparable to other burn survivor collectives. Sheckter et al. reviewed 493 adult burn survivors with a median age 46, mean %TBSA of 14, and 72% males [14], and Grant et al. compared burn survivors with (age 42, 69% male, 21% TBSA) and without (age 48, 67% male, 18% TBSA) problematic substance abuse [15]. The collective reviewed by Palmu et al. had similar mean age, gender distribution, and %TBSA. Likewise, injury mechanisms were comparable with flame burns being the most common cause [9]. Of note, the present study included only inpatient cases, but also those admitted for pain management primarily. It is possible that inclusion of patients treated in an outpatient setting would have resulted in even lower mean %TBSA.

HRQoL postburn was assessed using the SF-36 questionnaire. According to this, female patients scored significantly lower than men in 6 of 8 domains. This phenomenon has been observed before [16,17]. In 2018, Wasiak et al. exploited gender disparities in postburn HRQoL and found significantly lower SF-36 scores in women in the domains “physical functioning”, “mental health”, “vitality”, “social functioning”, and “general health” 12 months post injury [17]. This is consistent with the findings of our survey except we did not detect any differences concerning “social functioning” but for “role-physical” and “general health” instead, which were both lower in women. These results are surprising when considering that mean %TBSA was lower in women than in men. In line with our observations, female gender has been described as an independent risk factor for impaired HRQoL 12 months after burn injury [16]. When set in relation with current burns literature, SF-36 scores were comparably high in our collective. This may be due to the fact that the time from injury was relatively long (5 years on average) and that injuries were less severe when compared to existing literature [16,17].

Substance abuse and psychiatric diseases have been associated with prolonged and frequently complicated in-hospital treatment, as well as prolonged and less successful rehabilitation postburn [8,9,18,19,20]. Compared to published data, the prevalence of psychiatric comorbidities and substance-abuse disorders as documented in the hospital information system was fairly low. According to recent literature, the prevalence of psychiatric diseases among burn patients ranges between 15% and 55% [8,9], and the prevalence of substance-abuse disorders may be as high as 27% [9,21]. We found documented psychiatric comorbidities in 6%, whereas smoking, alcohol abuse, and drug abuse were described in 10%, 3%, and 2% of our patients, respectively. Since almost 40% of study participants reported they were regular smokers at the time of injury, this leads to the assumption that substance-abuse disorders and perhaps also psychiatric comorbidities were not adequately represented by clinical data.

As assessed via questionnaire, no significant differences were found for living situation before and after the burn injury, although five participants had moved from a rented to an owned place of residence. The postburn living situation showed no correlation with SF-36 general health scores, although mean values were higher in participants living in owned apartments or houses. None of the study participants lived in care facilities or depended on assisted living, meaning that all the included study participants returned to autonomous life after the injury. To the best of our knowledge, this finding is a novelty in burns literature.

When comparing relationship status of burn patients pre- and postburn, no significant changes were noted. However, one patient had widowed after injury, and fewer patients were in a relationship at the time of interrogation when compared to their preburn situation. Two-thirds of all participants were in a romantic relationship at the time of interrogation. This is consistent with the findings presented by Ohrtman et al., who found that 64% of burn survivors were in a romantic relationship and that this was equal to the rate of the general population. Injury characteristics, such as burn size or certain affected body areas or time since injury had no influence on relationship status [22]. Similarly, higher SF-36 general health scores were not predictive of patients being in a relationship postburn.

A slight, non-significant decrease in patient-reported tobacco consumption was noted after the burn injury. Almost half of participants claimed they were active smokers at the time of injury, which is relatively high when compared to existing literature [10]. This rate had decreased to approximately 40% postburn. Similar to smoking habits, fewer participants claimed to consume illicit drugs postburn. Mean SF-36 general health scores did not differ between smokers and non-smokers or drug users and non-users after the injury. Although the decrease in tobacco and drug abuse was not significant and no causal relation could be established with the data assessed, it is possible that the impact of the burn injury on the individual health situations motivated patients to a healthier lifestyle postburn.

Patient-reported alcohol consumption had decreased significantly when comparing pre- and postinjury values. Surprisingly, mean SF-36 general health scores were significantly higher in patients reporting high levels of alcohol consumption postburn. This is striking, when bearing in mind that recent evidence found a close correlation between alcohol consumption and reduced HRQoL in patients with alcohol use disorder [23]. Furthermore, Palmu et al. described a significant correlation between psychiatric diseases and alcohol dependence among burn victims [10]. To rule out any bias in our cohort, backwards linear regression analysis was performed: only male gender and high SF-36 general health scores remained as significant confounders for higher post-injury alcohol consumption, whereas injury characteristics, patient age, and psychiatric comorbidities were irrelevant. Further analysis of our cohort revealed that SF-36 general health scores were significantly lower in patients with documented pre-existing alcohol abuse. Based on these findings, two vastly differing assumptions can be made: either patients with pre-existing alcohol abuse did not truthfully declare their alcohol consumption, or—less likely —presence of inversed causality bias, meaning that SF-36 general health scores were higher because of higher alcohol consumption.

There is limited data available in medical literature concerning alcohol and drug abuse in burn survivors, although preburn substance-abuse disorders have been associated with impaired outcome on multiple occasions [15,24,25].

### Strengths and Limitations

To the best of our knowledge, this is the first study to compare patient-reported living situation, relationship status, and substance abuse pre- and postburn. The fact that all patients were interviewed by the same person and unclear questions could be explained are certainly strengths of the study.

The fact that all clinical data was collected in a retrospective rather than in a prospective fashion is certainly a strong limitation. Furthermore, the telephone survey was carried out during the COVID-19 pandemic. It is possible that the participants’ statements on HRQoL were influenced by intermittently necessary lockdowns with concomitant social isolation.

## 5. Conclusions

Based on the observations of this study, it can be concluded that HRQoL and social reintegration of our study respondents is acceptable. Postburn, a reduction in tobacco and alcohol consumption as well as illicit drug use was noted in the present study collective. At the same time, living situations and relationship status did not change significantly after the burn injury. More than men, women seem to suffer from consequences of burn injuries with respect to HRQoL. Future research should exploit long-term outcome after burn injuries and social reintegration. Furthermore, special attention should be paid to substance abuse in burn survivors.

## Figures and Tables

**Table 1:** Baseline parameters. * = significant.

Parameter		Women	Men	Overall	*p*
No. of patients		35 (27.3%)	93 (72.7%)	128 (100%)	
Age (injury)		41.2 ± 16.3	39.5 ± 15.6	40.0 ± 15.7	0.594
Age (inquiry)		46.1 ± 16.4	44.7 ± 16.2	45.1 ± 16.2	0.662
Month since injury		58.9 ± 26.5	62.0 ± 25.1	61.1 ± 25.4	0.538
%TBSA		5.6 ± 7.3	10.6 ± 11.8	9.2 ± 11.0	0.005 *
3rd degree injury		15 (42.9%)	51 (54.8%)	66 (51.6%)	0.227
Affected body region	Upper extremity	17 (48.6%)	65 (69.9%)	82 (64.1%)	0.025 *
	Lower extremity	23 (65.7%)	45 (48.4%)	68 (53.1%)	0.080
	Back	4 (11.4%)	13 (14.0%)	17 (13.3%)	0.705
	Anterior trunk	8 (22.9%)	22 (23.7%)	30 (23.4%)	0.924
	Genitals	1 (2.9%)	5 (5.4%)	6 (4.7%)	0.548
	Hands	9 (25.7%)	44 (47.3%)	53 (41.4%)	0.024 *
	Head/neck	11 (31.4%)	52 (55.9%)	63 (49.2%)	0.014 *
Inhalation injury		0	5 (5.4%)	5 (3.9%)	0.162
ABSI score		5 (4–6)	5 (3–6)	5 (3.5–6)	0.021 *
No. of surgeries		1 (0–1)	1 (0–1)	1 (0–1)	0.490
ICU stay		2 (5.7%)	22 (23.7%)	24 (18.8%)	0.020 *
LOS		12.9 ± 11.2	16.4 ± 17.9	15.5 ± 16.4	0.285
Circumstances	Work-related	8 (22.9%)	43 (46.2%)	51 (39.8%)	0.011 *
	Household	21 (60.0%)	74 (50.5%)	68 (53.1%)	
	Traffic	2 (5.7%)	3 (3.2%)	5 (3.9%)	
	Self-inflicted	1 (2.9%)	0	1 (0.8%)	
	Assault	1 (2.9%)	0	1 (0.8%)	
	Other	2 (5.7%)	0	2 (1.6%)	
Mechanism	Flame	15 (42.9%)	56 (60.2%)	71 (55.5%)	0.292
	Scald	14 (40.0%)	26 (28.0%)	40 (31.3%)	
	Contact	3 (8.6%)	4 (4.3%)	7 (5.5%)	
	Chemical	3 (8.6%)	4 (4.3%)	7 (5.5%)	
	Electric	0	3 (3.2%)	3 (2.3%)	

**Table 2:** Substance abuse and psychiatric preconditions according to retrospective analysis. * = significant.

Precondition	Women	Men	Overall	*p*
Psychiatric disease	6 (17.1%)	2 (2.2%)	8 (6.3%)	0.002 *
Smoking	3 (8.6%)	10 (10.8%)	13 (10.2%)	0.716
Alcohol abuse	3 (8.6%)	1 (1.1%)	4 (3.1%)	0.030 *
Drug abuse	2 (5.7%)	0	2 (1.6%)	0.020 *

**Table 3:** Relationship status, living situation, smoking, alcohol consumption, and substance abuse pre- and postburn. * = significant.

Parameter		Preburn	Postburn	*p*
Relationship status	married	54 (42.2%)	53 (41.4%)	0.415
	relationship, not married	43 (33.6%)	33 (25.8%)	
	single	28 (21.9%)	38 (29.7%)	
	widowed	3 (2.3%)	4 (3.1%)	
Living situation	owned house/apartment	76 (59.4%)	81 (63.3%)	0.521
	rented house/apartment	52 (40.6%)	47 (36.7%)	
Smoking	>1 pack/week	49 (38.8%)	40 (31.3%)	0.130
	<1 pack/week	11 (8.6%)	7 (5.5%)	
	never	68 (53.1%)	81 (63.3%)	
Alcohol consumption	never	23 (18.0%)	35 (27.3%)	0.019 *
	1 ×/month	21 (16.4%)	23 (18.0%)	
	2–4 ×/month	41 (32.0%)	41 (32.0%)	
	2–3 ×/week	33 (25.8%)	25 (19.5%)	
	>4 ×/week	10 (7.8%)	4 (3.1%)	
Drug abuse	no	118 (92.2%)	121 (94.5%)	0.451
	yes	10 (7.8%)	7 (5.5%)	

**Table 4:** SF-36 general health scores relative to living situation, relationship, smoking, alcohol consumption, and drug abuse postburn. * = significant.

Postburn		Patients	Mean SF-36 General Health	*p*
Relationship	married/in relationship	86 (67.2%)	82.0 ± 24.0	0.517
	single/widowed	42 (32.8%)	78.9 ± 26.8	
Living situation	owned	81 (63.3%)	84.0 ± 23.7	0.076
	rented	47 (36.7%)	75.9 ± 26.3	
Smoking	no	81 (63.3%)	79.1 ± 26.1	0.258
	yes	47 (36.7%)	84.3 ± 22.5	
Alcohol consumption	low	99 (77.3%)	77.9 ± 27.0	<0.001 *
	high	29 (22.7%)	91.6 ± 10.6	
Drug abuse	no	121 (94.6%)	88.6 ± 9.9	0.094
	yes	7 (5.4%)	80.5 ± 25.4	

## Data Availability

The data presented in this study are available on request from the corresponding author. The data are not publicly available due to data protection.

## Supplementary Materials

The following supporting information can be downloaded at: https://www.mdpi.com/article/10.3390/medicina58050563/s1, Table S1: Questionnaire.
