# Supplementary material for: Life after Burn, Part II: Substance Abuse, Relationship and Living Situation of Burn Survivors
Source: Medicina (Kaunas). 2022 Apr 19;58(5):563. doi: 10.3390/medicina58050563 (PMC9147374; doi:10.3390/medicina58050563)
Supplement: Supplementary file 1 [file medicina-58-00563-s001.zip › medicina-1642973-supplementary.pdf]

**Table S1**. Questionnaire.

| Have you been in a relationship before your burn injury? | Are you in a relationship at present? |
|---|---|
| a. yes, married | a. yes, married |
| b. yes, not married | b. yes, not married |
| c. no, single | c. no, single |
| d. no, divorced | d. no, divorced |
| **How did you live before your burn injury?** | **How do you live at present?** |
| a. owned apartment/house | a. owned apartment/house |
| b. rented apartment/house | b. rented apartment/house |
| c. assisted living | c. assisted living |
| d. care facility | d. care facility |
| **How often did you drink alcohol before your burn injury?** | **How often do you drink alcohol at present?** |
| a. never | a. never |
| b. once a month | b. once a month |
| c. 2-4 times a month | c. 2-4 times a month |
| d. 2-3 times a week | d. 2-3 times a week |
| e. 4 times or more a week | e. 4 times or more a week |
| **Did you smoke before your burn injury?** | **Do you smoke at present?** |
| a. yes (more than one pack a week) | a. yes (more than one pack a week) |
| b. occasionally (less than one pack a week) | b. occasionally (less than one pack a week) |
| c. never | c. never |
| **Did you consume drugs before your burn injury?** | **Do you take drugs regularly at present?** |
| a. yes | a. yes |
| b. no | b. no |
